# Supplementary material for: High throughput barcoding method for genome-scale phasing
Source: Sci Rep. 2019 Dec 2;9:18116. doi: 10.1038/s41598-019-54446-x (PMC6889410; doi:10.1038/s41598-019-54446-x)
Supplement: Supplementary file 1 — SI [file 41598_2019_54446_MOESM1_ESM.pdf]

SUPPLEMENTARY INFORMATION

High throughput barcoding method for genome-scale phasing

David Redin[1], Tobias Frick[1], Hooman Aghelpasand[1], Max Käller[1], Erik Borgström[1], Remi-Andre Olsen[2], and Afshin Ahmadian[1,*]

[1] Royal Institute of Technology (KTH), School of Engineering Sciences in Chemistry, Biotechnology and Health, Department of Gene Technology, Science for Life Laboratory, SE-171 65, Solna, Sweden.

[2] Stockholm University, Department of Biochemistry and Biophysics, Science for Life Laboratory, Box 1031, 171 21 Solna, Sweden.

* Correspondence and requests for materials should be addressed to A.A. (email: afshin.ahmadian@scilifelab.se).

Table of Contents

Supplementary Figure S1 ____________________________ p.2

Supplementary Figure S2 ____________________________ p.3

Supplementary Figure S3 ____________________________ p.4

Supplementary Figure S4 ____________________________ p.5

Supplementary Figure S5 ____________________________ p.6

Supplementary Figure S6 ____________________________ p.7

Supplementary Table S1 _____________________________ p.8

Supplementary Table S2 _____________________________ p.9

Supplementary Table S3 _____________________________ p.10

Supplementary Table S4 _____________________________ p.11

Supplementary Table S5 _____________________________ p.12

Supplementary Table S6______________________________p.13

Supplementary Note ________________________________ p.14

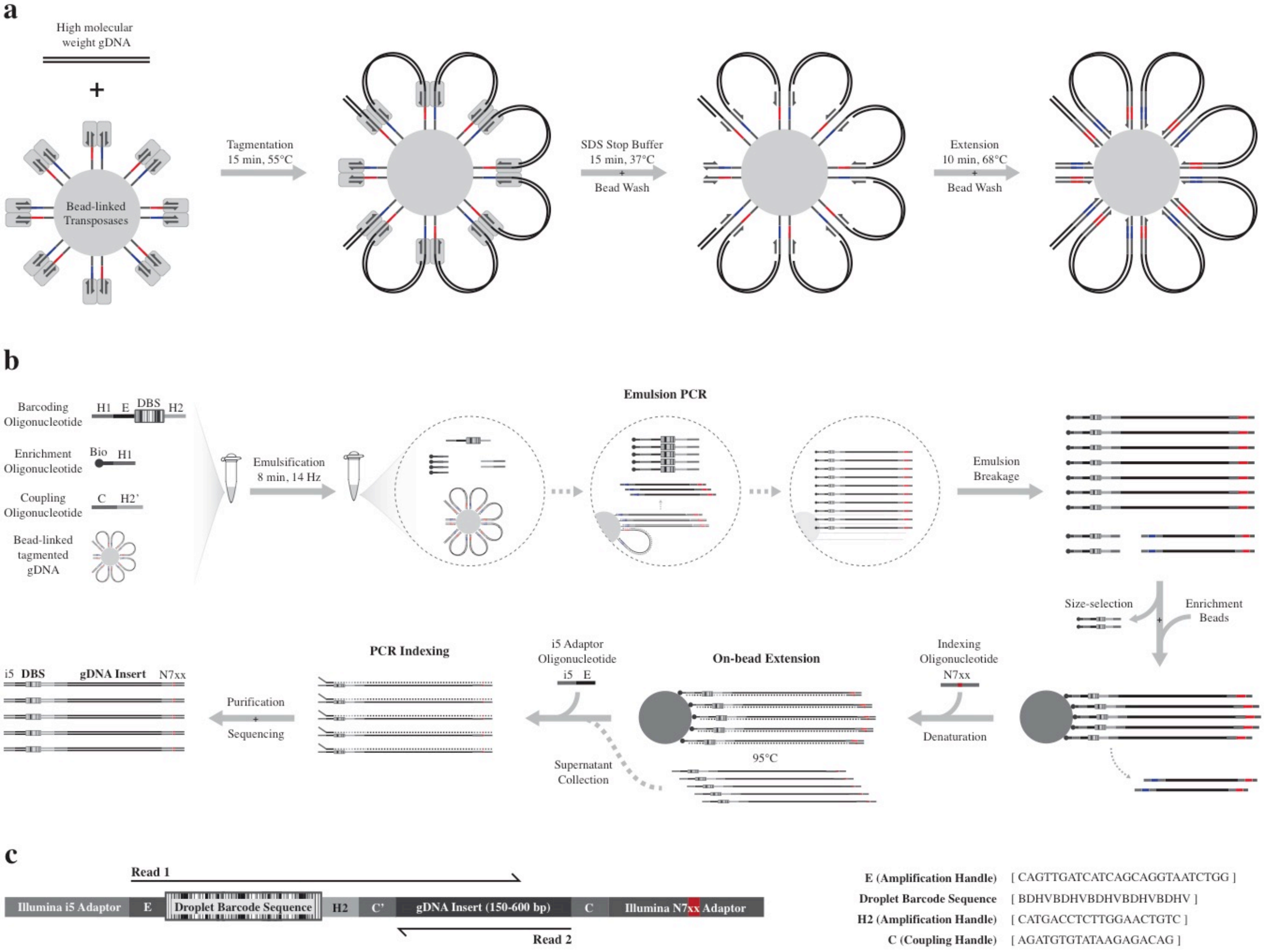

Supplementary Figure S1. Detailed method overview illustrating bead-bound template preparation (a), subsequent assay protocol (b) and sequencing read layout (c). For clarity, only the contents of a single droplet and the manipulation of corresponding product molecules are shown in the figure. The protocol for (a) follows User Guidelines for the Nextera DNA Flex Library Preparation kit, with the exception that an extension is performed without added oligonucleotides instead of the specified PCR cycling protocol. The process of tagmentation introduces a pair of universal sequences (shown in red and blue) at multiple random positions over the length of long DNA fragments. (b) Bead-bound template molecules and assay oligonucleotides (Supplementary Table 6) are incorporated into emulsion droplets by shaking. Inside the droplets, clonal amplification of the barcoding molecule (featuring DBS, a Droplet Barcode Sequence, with a degeneracy of 3^20, or ~3.5*10^9) is performed asymmetrically to yield single stranded products, followed by coupling to the universal sequences on template molecules. After emulsion breakage, uncoupled amplicons are removed through bead-assisted size selection, and then by streptavidin-biotin based enrichment. An on-bead extension is performed and the supernatant is collected after denaturing to prevent uncoupled barcoding molecules from interfering with the indexing. Finally, an i5 sequencing adaptor is added by PCR for sequencing using the Illumina platform. (c) After sequencing, the reads are trimmed and clustered according to the droplet barcode sequence to form barcoded read groups (Supplementary Figure 4). Note, the lengths of adapters, handles and genomic information are not drawn to scale.

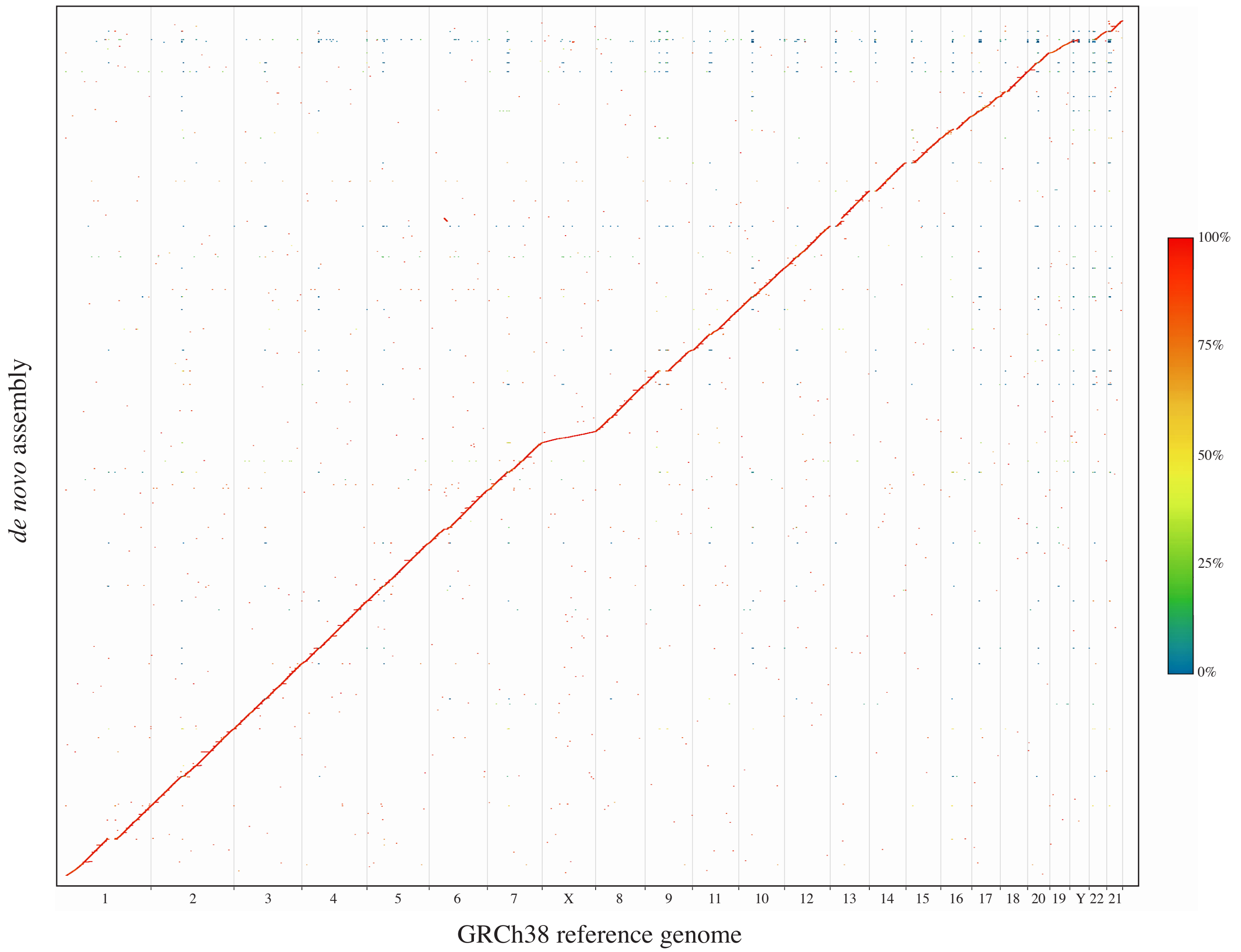

Supplementary Figure S2. Dot plot of GM24385 *de novo* assembly against the GRCh38 reference genome. Data is shown as a heatmap of the mean percent identity (per query), for a minimum query length of 1 kb and excluding scaffolds <10 kb. The reference is divided in the order of descending chromosome size. A lower assembly contiguity can be observed in chrX and chrY but such discrepancies are to be expected since these chromosomes are harder to assemble. The total length of the assembly was 3.20 Gb (2.68 Gb ungapped), covering 2.65 Gb (85.4%) of the 3.10 Gb GRCh38 reference. For comparison, an external 10x Genomics assembly showcased online made from 1.19 B reads (58X) yielded an assembly length of 2.88 Gb (2.82 Gb ungapped) with an N50 scaffold length of 36.5 Mb.

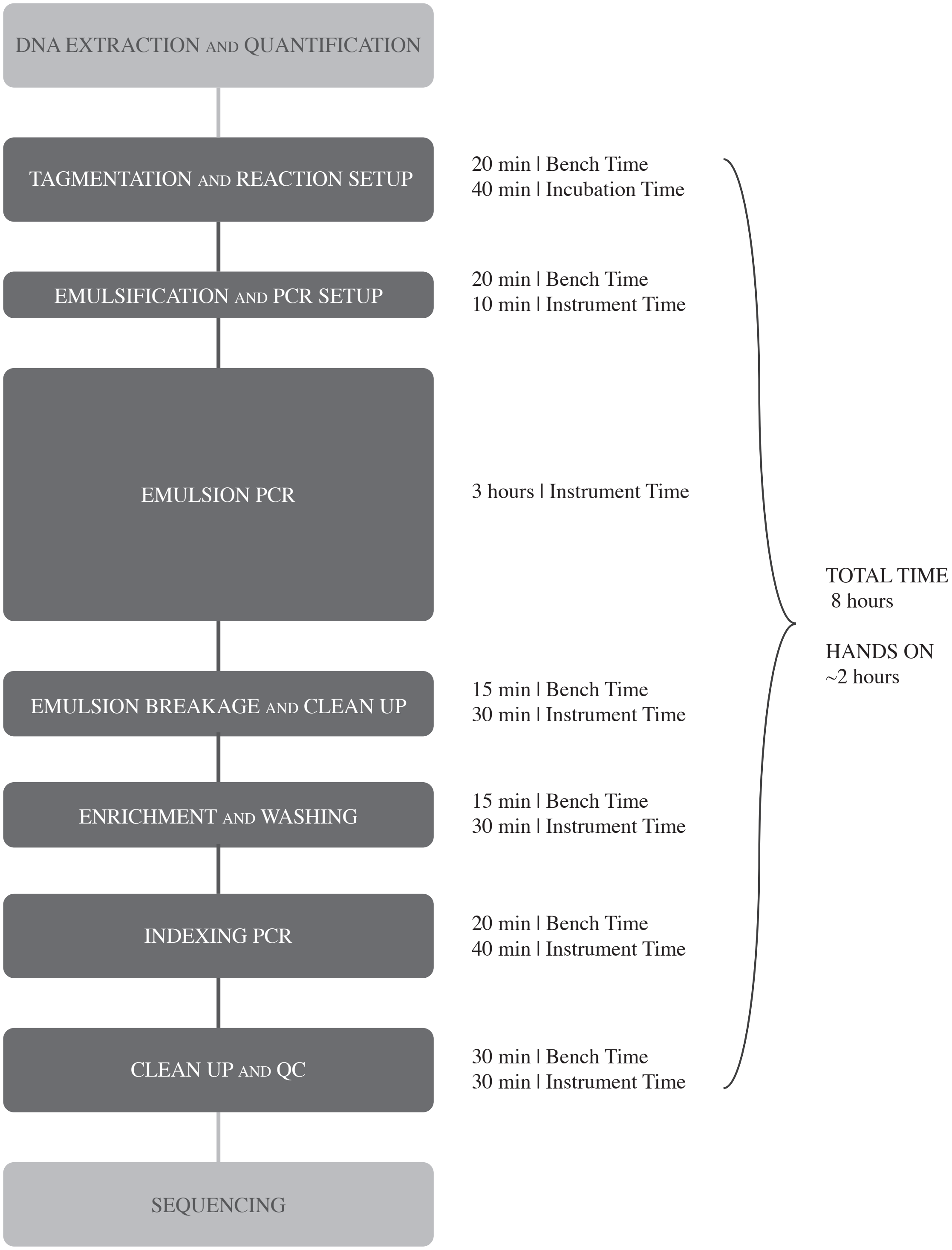

Supplementary Figure S3. Overview of protocol steps with corresponding bench and instrument times. The whole protocol can be performed in a single day, excluding DNA extraction and DNA sequencing.

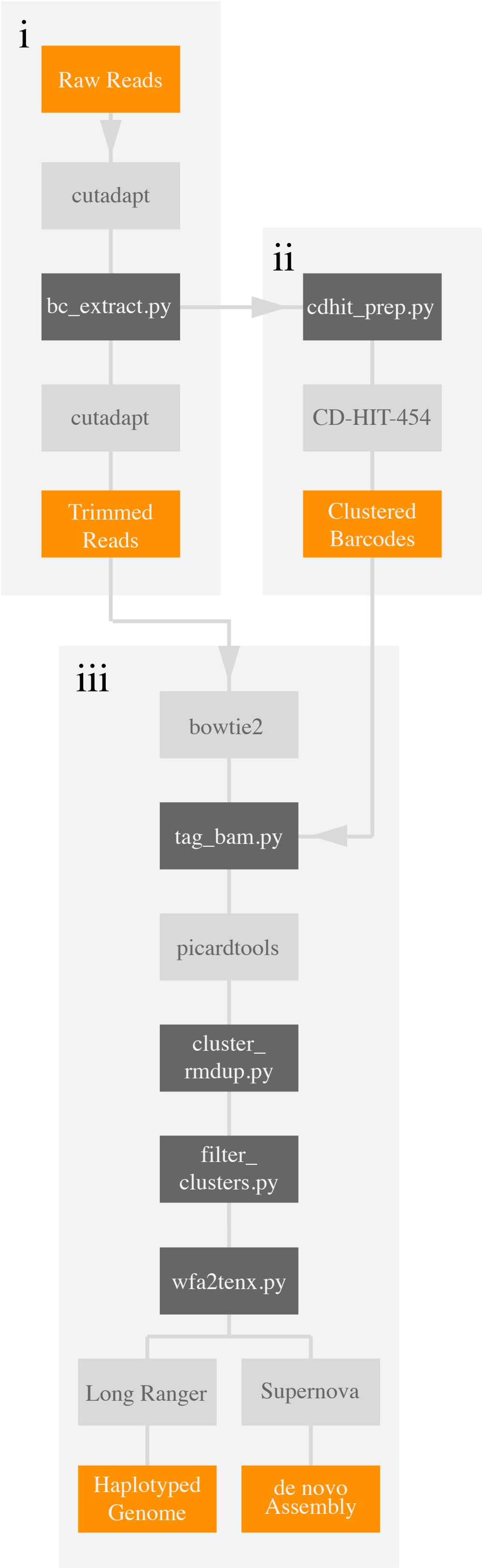

Supplementary Figure S4. Flowchart of computational pipeline for steps (i) Read trimming, (ii) barcode clustering and (iii) filtering, followed by human phasing and *de novo* pipelines. In-house developed scripts are marked in dark grey.

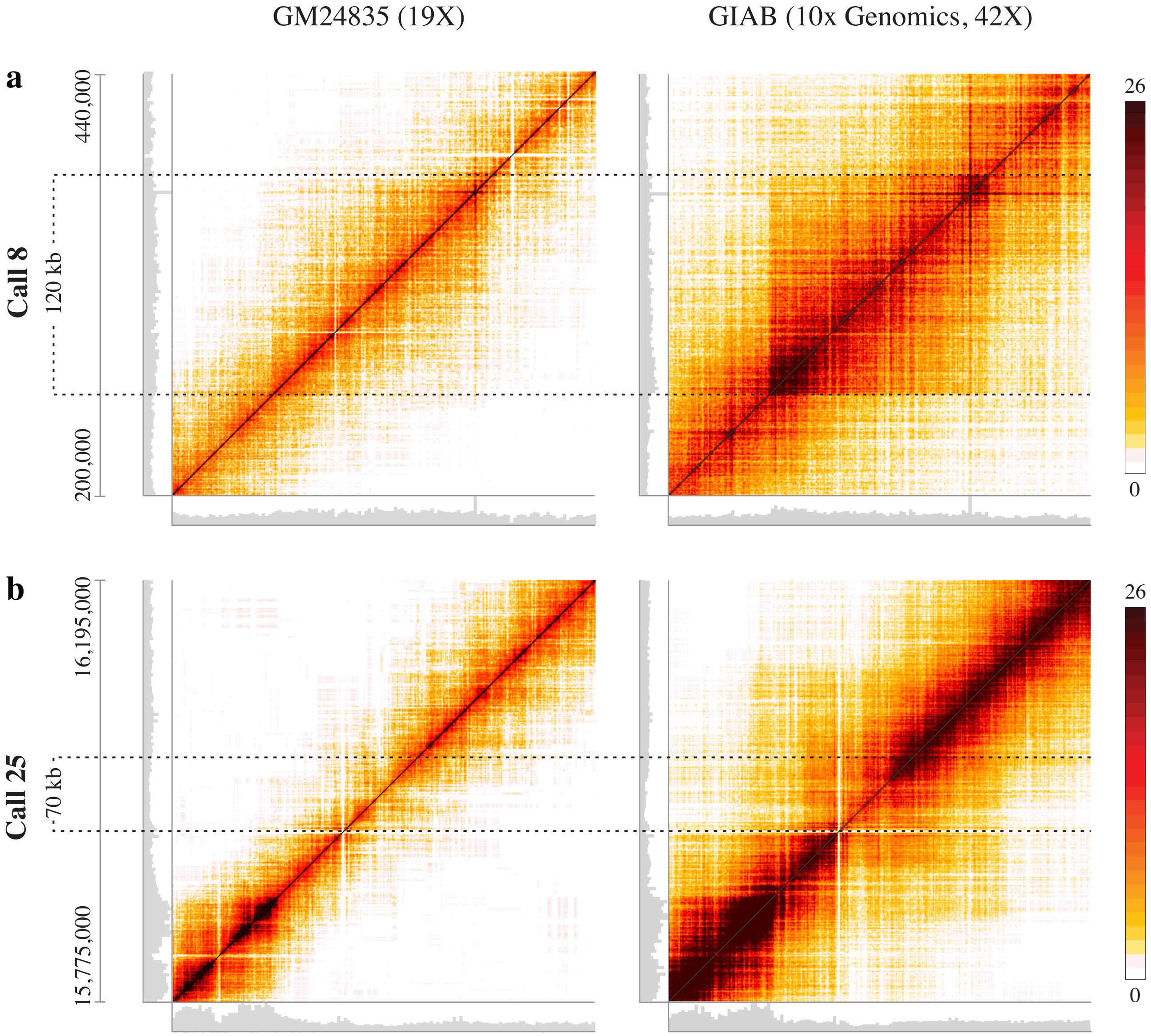

Supplementary Figure S5. Visual inspection of barcode overlaps in the GIAB (10x Genomics, 42X) reference dataset, with the coordinates corresponding to (a) variant call 8 and (b) variant call 25 in the GM24385 (19X) dataset shown by dotted lines. See Supplementary Table 3 for variant call specifications. Shown in (a), variant call 8 is a duplication event spanning 120 kb on chromosome 6, wherein the barcode overlap and coverage of the reference dataset suggests the presence of a localized duplication event despite it not being called by the software for this dataset. Similarly for (b), there is a localized decrease in coverage within the reference dataset that supports the validity of variant call 25 which has exclusively been called in the GM24385 (19x) dataset.

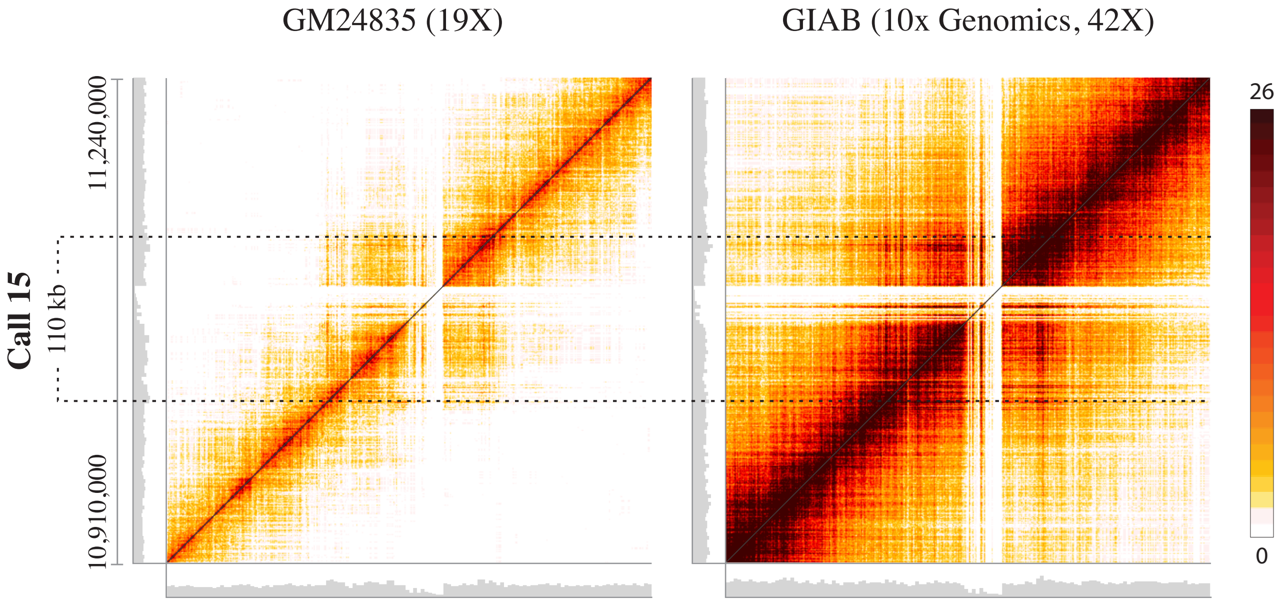

Supplementary Figure S6. Visual inspection of barcode overlapping for non-verified variant call 15, corresponding to GM24385 (19 X) (left) and GIAB (10x Genomics, 42X) reference dataset (right). The region covered by the identified variant in chromosome 12 (duplication event spanning 120 kb) features a significant drop out in coverage that has likely influenced the accuracy by which variants can be called. Since the same pattern of non-existent barcode overlap is observed for the reference dataset, the authenticity of the variant call could not be verified.

Supplementary Table S1. Assay cost assessment.

**Reagent**	**Supplier**	**Cost Per Library**
Nextera DNA Flex Library Prep Kit *	Illumina	$5.83
Phusion® Hot Start Flex 2X Master Mix	NEB	$3.62
Emulsion Additives	Sigma	$0.04
Oligonucleotides	IDT	$0.05
Novec 7500 with 5%wt 008-FS	RAN Biotechnologies	$3.78
FC-40 with 5%wt 08-FS	RAN Biotechnologies	$2.76
1*H*,1*H*,2*H*,2*H*-Perfluoro-1-octanol	Sigma	$1.22
DynaBeads MyOne Streptavidin T1	Thermo Scientific	$1.65
**Total ** **		**$18.95**

* Based on reagent volumes provided in Nextera DNA Flex Library Preparation Kit for 24 reactions, of which 15% of a single reaction volume was used per assay library.

** Costs for reagents have been converted from SEK to USD using an exchange rate of 0.116 USD/SEK. Common materials (plastic consumables and buffers) have not been included in the cost assessment.

Supplementary Table S2. Processing of reads for GM24385 (19 X). The number of sequencing reads (not paired) and the corresponding number of barcoded read groups are detailed after removal of reads within the analysis pipeline, as described in Methods and Supplementary Figure 4. A total of 233,006,619 reads (25.8%) were marked as duplicates.

Analysis Step	Reads Remaining	% Total	Barcoded Read Groups
Raw data	903,723,210	100%	3,339,866
Trimming	890,047,118	98.5%	3,339,866
Filtering	641,457,522	71.0%	2,204,497

Supplementary Table S3. Verification of large structural variant (LSV) calls in GM24385 (19X) against the GIAB (10x Genomics, 42X) resource dataset. Phasing analysis was performed with the same reference and version of the Long Ranger pipeline for both datasets. In addition to specified LSV calls, multiple heterozygous deletions were called in the X chromosome for both datasets, despite the individual in question being a male. These anomalies were reported and subsequently confirmed as erroneous by 10x Genomics.

		**GM243825 (19 X)**			**GIAB (10x Genomics)**			
Call	Chr	Position	Type	Size	Position	Type	Size	Note
1	1	72,300,641 - 72,346,143	DEL	45.5 kb	72,300,641 - 72,346 200	DEL	45.6 kb	
2	1	121,610,000 - 121,940,000	DEL	330 kb	121,610,000 - 121,840,000	DEL	230 kb	
3	2	34,470,699 - 34,511,486	DEL	40.8 kb	34,470,693 - 34,511,500	DEL	40.8 kb	
4	3	130,044,539 - 130,088,085	DEL	43.5 kb	130,044,542 - 130,087,886	DEL	43.3 kb	
5	4	34,778,034 - 34,827,542	DEL	49.5 kb	34,778,232 - 34,827,339	DEL	49.5 kb	
6	4	68,508,038 - 68,625,387	DEL	117 kb	68,508,058 - 68,625,383	DEL	117 kb	
7	4	69,270,000 - 69,370,000	DEL	100 kb	69,280,000 - 69,370,000	DEL	90 kb	
8	6	260,000 - 380,000	DUP	120 kb	N/A	Visually confirmed		***
9	6	32,483,200 - 32,586,623	DUP	103 kb	32,483,184 - 32,591,863	UNK	109 kb	*
10	7	143,127,754 - 143,196,851	DEL	69.1 kb	143,127,755 - 143,196,800	DEL	69.0 kb	
11	9	60,550,000 - 60,630,000	DEL	80.0 kb	60,550,000 - 60,630,000	DEL	80.0 kb	
12	10	41,864,883 - 41,904,216	UNK	39.3 kb	41,859,660 - 41,900,226	UNK	40.6 kb	
13	11	55,590,000 - 55,660,000	DEL	70.0 kb	55,597,954 - 55,660,000	UNK	80.4 kb	*
14	12	17,767,858 - 17,853,857	INV	86.0 kb	17,773,181 - 17,869,963	INV	96.8 kb	
15	12	11,020,000 - 11,130,000	DUP	110 kb	N/A	Not verified		****
16	12	11,084,352 - 11,126,407	INV	42.1 kb	11,083,875 - 11,126,374	UNK	42.5 kb	*
17	14	19,860,000 - 19,950,000	DUP	90.0 kb	19,700,000 - 19,950,000	DUP	225 kb	
18	14	105,770,000 - 106,420,000	DEL	650 kb	105,760,000 - 106,630,000			
19	14	106,420,000 - 106,540,000	DEL	120 kb	105,760,000 - 106,630,000	DEL	870 kb	**
20	14	106,540,000 - 106,630,000	DEL	90.0 kb	105,760,000 - 106,630,000			
21	16	22,610,000 - 22,700,000	DUP	90.0 kb	22,530,000 - 22,700,000	DUP	170 kb	
22	16	34,040,000 - 34,240,000	DUP	200 kb	34,110,000 - 34,210,000	DUP	100 kb	
23	17	46,140,000 - 46,240,000	DUP	100 kb	46,140,000 - 46,230,000	DUP	90 kb	
24	19	24,330,000 - 24,410,000	DUP	80.0 kb	24,330,000 - 24,410,000	DUP	80.0 kb	
25	22	15,950,000 - 16,020,000	DEL	70.0 kb	N/A	Visually confirmed		***
26	22	22,760,000 - 22,910,000	DEL	150 kb	22,758,960 - 22,907,024	DEL	148 kb	
27	Y	9,470,000 - 10,250,000	DEL	780 kb	9,460,000 - 10,260,000	DEL	800 kb	
28	Y	18,850,000 - 20,070,000	DEL	1.22 Mb	10,600,000 - 20,070,000	DEL	9.47 Mb	
29	Y	20,350,000 - 21,490,000	DEL	1.14 Mb	20,350,000 - 26,630,000	DEL	6.28 Mb	

* Variant type not classified in the GIAB (10x Genomics) dataset.
** Adjoining calls identified as a single variant in the GIAB (10x Genomics) dataset.
*** Variant call visually supported from barcode overlap and read mappings (Supplementary Figure 5)
**** Visual inspection does not support verification of the variant call (Supplementary Figure 6).

Supplementary Table S4. Method reproducibility. To investigate the variability of the method a replicate experiment was performed using the same protocol for which the metrics supplied from the Long Ranger report is shown below.

Library	GM24385 (19X)	Replicate
Sequencing reads	641,457,522	656,818,584
Mean depth	19.1 X	21.6 X
SNPs Phased	97.9%	98.4%
N50 Phase Block	1,832,815 bp	1,580,611 bp
Longest Phase Block	7,771,012 bp	10,473,271 bp
Mean Molecule Length	25,946 bp	41,075 bp
Molecules >20 kb	74.8%	81.7%
Molecules >100 kb	18.6%	12.0%
LSV Calls *	35	33
Short Deletion Calls	4,008	3,616
Median Insert Size	231 bp	273 bp
Mapped Reads	82.7%	89.6%
Zero Coverage	0.735%	0.708%
Q30 bases, Read 1	82.4%	87.8%
Q30 bases, Read 2	64.2%	75.0%

* Variants where verified using the same workflow as those presented in Supplementary Table S3, where this library had 6 visually confirmed variants.

Supplementary Table S5. Single Nucleotide Variation (SNV) call counts and raw comparison to a the GIAB 'ground truth' callset for GM24385.

Library	GM24385 (19X)	GM24385 (35X)	GIAB (10x Genomics, 42X)
Sequencing Depth	19.1X	34.7 X	41.7 X
Total SNV Calls	3,697,907	3,924,654	4,517,053
SNVs (phred score >60)	3,445,072	3,804,691	4,054,372
True Positive SNV Calls	3,287,856	3,599,024	3,675,500
Non-called SNVs	1,031,533	720,359	643,885
False Positive SNV Calls	157,210	205,655	378,862
Discordant SNV Calls	6	12	10

Supplementary Table S6. Oligonucleotide sequences.

Name	Sequence (5' - 3')
Enrichment Oligo	BIOTIN-GGAGCCATTAAGTCGTAGCT
Barcoding Oligo	GGAGCCATTAAGTCGTAGCTCAGTTGATCATCAGCAGGTAATCTGG BDHVBDHVBDHVBDHVBDHVCATGACCTCTTGGAACTGTC
Coupling Oligo	CTGTCTCTTATACACATCTGACAGTTCCAAGAGGTCATG
Indexing Oligo *	CAAGCAGAAGACGGCATACGAGAT [8 bp Index] GTCTCGTGGGCTCGG
i5 Adaptor Oligo	AATGATACGGCGACCACCGAGATCTACACTCTTTCCCTACACGACGCT CTTCCGATCTCAGTTGATCATCAGCAGGTAATCTGG

* The indexing oligonucleotide is identical to Illumina's N7xx Adapters from the Nextera XT Index Kit v2.

Supplementary Note. Coupling efficiency.

Based on mapping positions identified within the barcoded read groups, molecule lengths were defined as the distance spanning the outermost reads. Given the reads spanning each molecule, a measure of bases sequenced were calculated for each molecule, yielding an average coverage of 18.6%.

Given transpose adaptors A and B, the template molecules formed will be structured as either A-A', A-B', B-A', or B-B'. As a consequence of this, half of the template molecule will be unavailable for amplification due to complementary ends forming secondary hairpin structures (A-A' and B-B'). Furthermore, given the protocol used for library indexing (Supplementary Figure 1), only half of the available molecules will be amplified to yield products for sequencing. The coupling efficiency of barcode sequences to template molecules can thus crudely be appreciated to be 74.4% (18.6 * 2 * 2).
